# Endoscopic Management of Delayed Presentation of Iatrogenic Type 1 Duodenal Perforation Using Off-Label Esophageal Fully Covered Self-Expandable Metal Stent

**DOI:** 10.14309/crj.0000000000002238

**Published:** 2026-07-10

**Authors:** Herisha Shah, Tushaar Shrimanker, Syed Hamza Sohail, John Miller, Nha Tri Duong

**Affiliations:** 1 Department of Internal Medicine, University of Massachusetts Chan Medical School-Baystate, Springfield, MA; 2 Division of Gastroenterology, University of Massachusetts Chan Medical School-Baystate, Springfield, MA

**Keywords:** duodenal perforation, ERCP, advanced endoscopy

## Abstract

We present the case of a 75-year-old woman with pancreatic cancer who presented with a type 1 duodenal perforation (large lateral wall perforation) following endoscopic retrograde cholangiopancreatography for palliative biliary stent replacement. Owing to prohibitive operative risk and based on multispecialty input, surgical intervention was deferred in favor of a salvage, off-label, endoscopic deployment of an esophageal fully covered self-expandable metal stent to traverse the area of perforation and facilitate healing by secondary intention, which was ultimately successful. This case demonstrates the feasibility of a nonoperative approach in the management of duodenal perforations in high-risk surgical candidates.

## INTRODUCTION

Endoscopic retrograde cholangiopancreatography (ERCP) is the workhorse of endoscopic therapy for pancreaticobiliary disease that can have notable complications, including postprocedural pancreatitis, perforation, cholangitis, cholecystitis, bleeding, and sedation-related side effects.^1^ This report focuses primarily on duodenal perforation, a rare but serious complication of ERCP with an incidence of 0.08%-0.6% in the general population and a mortality rate of 8%-23%.^2,3^ Concurrent pancreatic malignancy may predispose patients to perforation from duodenal angulation and/or stricture, focal compromise of tissue integrity, and greater procedure length due to technical difficulty.

Stapfer et al categorized ERCP-related duodenal perforations into 4 types based on the location, severity, and mechanism of injury to dictate management (Table 1).^1,4^

**Table 1.** Types of endoscopic retrograde cholangiopancreatography-related duodenal perforations as per Stapfer classification

Type	Location
Type 1	Perforation of the lateral or medial wall of the duodenum
Type 2	Periampullary injuries/often seen after sphincterotomy
Type 3	Distal bile duct or pancreatic duct injuries
Type 4	Retroperitoneal microperforations from excessive insufflation

Traditionally, iatrogenic and traumatic duodenal perforations have been treated with early surgical repair, but recent advancements in endoscopic devices and techniques allow the advent of nonoperative early endoscopic management methods including the use of through-the-scope clips (TTSCs), over-the-scope clips (OTSCs), band ligation, endoloops, fibrin glue, and stents.^1,3,5^

Currently, there is a paucity of data to support the endoscopic management of duodenal perforations. This case demonstrates the use of a novel approach to manage a type 1 duodenal perforation endoscopically and the encountered considerations and drawbacks to this approach to help bolster the available data and inform decision making in future cases.

## CASE REPORT

**Figure 1.**
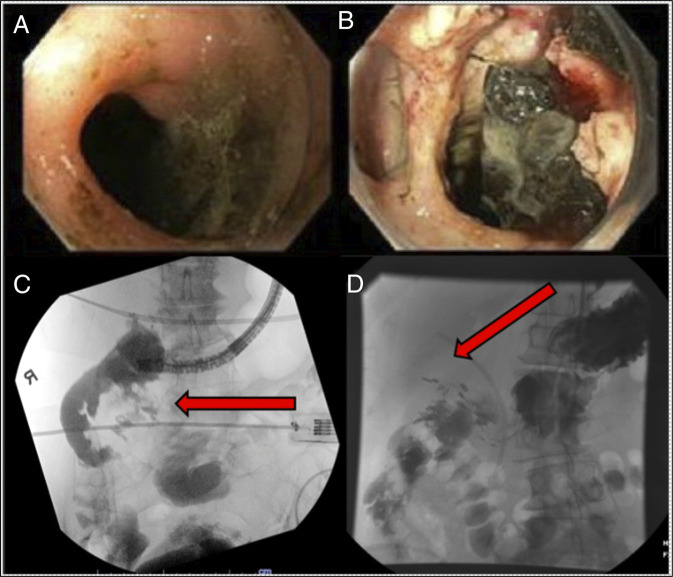
Duodenal perforation on imaging. (A) Distal duodenal bulb defect on EGD. (B) Necrotic debris seen through duodenal perforation within retroperitoneal collection on EGD. (C) Duodenal perforation and contrast extravasation on fluoroscopy (red arrow). (D) Duodenal perforation and contrast extravasation on fluoroscopy. EGD, esophagogastroduodenoscopy (red arrow).

**Figure 2.**
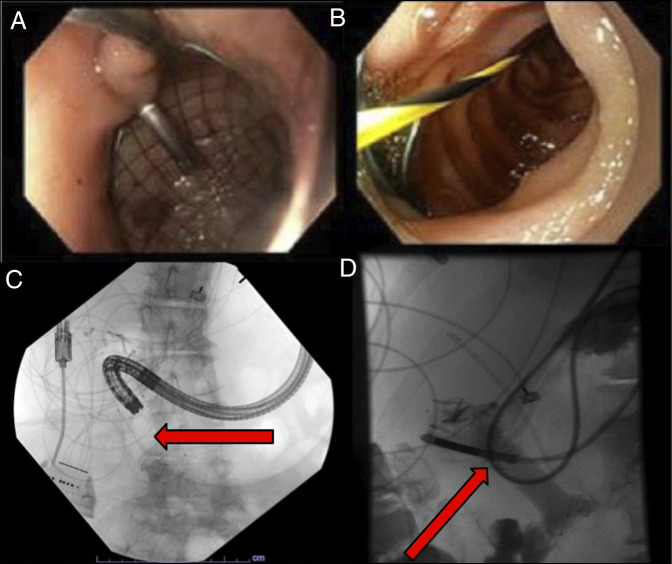
Esophageal FCSEMS on imaging. (A) Wire-guided insertion of esophageal FCSEMS on EGD. (B) Wire-guided insertion of esophageal FCSEMS on EGD. (C) Esophageal FCSEMS in duodenum on fluoroscopy (red arrow). (D) Esophageal FCSEMS in duodenum on fluoroscopy with contrast-opacifying stent (red arrow). EGD, esophagogastroduodenoscopy; FCSEMS, fully covered self-expandable metal stent.

A 75-year-old woman with pancreatic adenocarcinoma and prior biliary stent placements, diabetes, peripheral vascular disease, recent lower extremity dry gangrene, heart failure, and atrial fibrillation, was being managed for malignant distal biliary stricture at an outside center. ERCP was attempted for stent exchange, but a duodenal stricture prohibited scope passage, despite 20 mm balloon dilation of the stricture. She was discharged home, however presented to the hospital the following day with severe abdominal pain. Computed tomography imaging revealed a duodenal perforation with contained leak. Owing to poor clinical status, she was deemed high risk for operative repair and initially managed conservatively. Because of persisting leak, multidisciplinary consultation favored endoscopic therapy. Upper endoscopy showed a 4 cm full-thickness perforation at the D1/D2 junction on superior/anterior wall (Figure 1). Wire-guided placement of an 18 mm × 10 cm esophageal fully covered self-expandable metal stent (FCSEMS) was undertaken and fixed proximally using endoscopic suturing and TTSCs (Figure 2). A nasojejunal tube was placed for enteral feeding. Despite initial clinical improvement, imaging 2 weeks later revealed a persistent leak and abscess formation. Repeat endoscopy showed partial distal stent migration, likely due to duodenal angulation from malignant compression. A further 20 mm × 8 cm esophageal FCSEMS was deployed in a stent-in-stent manner with fixation using endoscopic tacking sutures and TTSCs. The leak resolved on imaging, and the patient tolerated oral intake.

Both stents were removed without complications 3 months later, and endoscopy revealed granulation tissue and ulceration without residual perforation. Same session ERCP (with forward-viewing therapeutic scope to better navigate anatomical distortion) allowed for successful exchange of the biliary stent with a covered metal biliary stent to palliate malignant distal biliary stricture.

## DISCUSSION

Type 1 duodenal perforations are typically characterized by sudden bleeding, luminal deflation, and difficulty maintaining insufflation.^5^ In patients with pancreatic and duodenal malignancy, extrinsic or intrinsic compression may distort anatomy and translate to intraprocedural challenges. Duodenal perforations often require prompt surgical intervention to achieve optimal clinical outcomes. Surgical options include primary repair of the perforation, drainage of an associated abscess or phlegmon, choledochojejunostomy, or pancreatoduodenectomy.

However, if early endoscopic closure appears feasible, surgery may be avoided. Large type 1 duodenal perforations (lateral wall tears >3 cm) are technically challenging to close endoscopically.^5^ By contrast, smaller defects can often be successfully managed using TTSCs, OTSCs, band ligation, or endoloops.^5^ For larger but contained defects, novel endoscopic suturing systems have expanded therapeutic options. Clinical success after endoscopic closure is reported in more than 90% of patients, with closure rates ranging from 88% to 100% for small perforations (less than 13 mm).^5^

Although conventional management discourages endoscopic intervention in delayed type 1 duodenal perforations (>24 hours post-onset), our high-risk surgical candidate was successfully treated with salvage placement of esophageal FCSEMS secured with proximal flange fixation. The patient's contained retroperitoneal collection was managed nonoperatively, and healing occurred without adjunctive percutaneous drainage.

Enteral stents may be considered in similar cases among patients who are poor surgical candidates. However, covered duodenal stents are currently unavailable in the United States. In such scenarios, off-label use of esophageal FCSEMS provides a practical alternative, with proximal stent fixation mitigating migration risk, as used in our patient. While this strategy achieved successful closure, a stent revision was later required due to partial migration. Our institutional experience since this index case suggests that migration remains a significant limitation, underscoring the need for multicenter data to better define the safety and efficacy of this approach. Weekly abdominal radiographs are encouraged to identify early migration.

In selected nonsurgical candidates with type 1 duodenal perforations, off-label placement of esophageal FCSEMS offers a viable, minimally invasive alternative when standard closure methods are impractical. Generally, we would expect adjuvant percutaneous drainage to be needed in such a case of delayed presentation of duodenal perforation, remarkably however, no percutaneous drainage was required in this case. Although not US Food and Drug Administration approved for this indication, esophageal FCSEMS can effectively serve as a substitute. Although migration remains a major risk, this may be mitigated through secure fixation or stent-in-stent techniques. Until dedicated covered duodenal stents become available, esophageal FCSEMS placement presents a viable option in complex, high-risk cases.

## DISCLOSURES

Author contributions: All authors have contributed equally. H. Shah, T. Shrimanker, SH Sohail were involved in writing and reviewing the manuscript. J. Miller and NT Duong were involved in helping with revisions and edits. J. Miller is the article guarantor.

Financial disclosure: None to report.

Previous presentation: An abridged version was previously presented at the American College of Gastroenterology Annual Scientific Meeting, October 24–29, 2025; Phoenix, AZ.

Informed consent was obtained for this case report.

## ABBREVIATIONS:

cm: centimeter(s)
D1/D2 Junction: Where the first part of the duodenum meets the second part of the duodenum
EGD: esophagogastroduodenoscopy
ERCP: endoscopic retrograde cholangiopancreatography
mm: millimeter(s)
OTSCs: over-the-scope clips
TTSCs: through-the-scope clips
